# Transcriptome analysis and characterisation of gill-expressed carbonic anhydrase and other key osmoregulatory genes in freshwater crayfish *Cherax quadricarinatus*

**DOI:** 10.1016/j.dib.2015.08.018

**Published:** 2015-09-02

**Authors:** Muhammad Yousuf Ali, Ana Pavasovic, Peter B. Mather, Peter J. Prentis

**Affiliations:** a School of Earth, Environmental and Biological Sciences, Queensland University of Technology, Brisbane, QLD 4001, Australia; b School of Biomedical Sciences, Queensland University of Technology, Brisbane, QLD 4001, Australia

**Keywords:** pH balance, Salinity, Redclaw, Crayfish, Transport-gene

## Abstract

The pH and salinity balance mechanisms of crayfish are controlled by a set of transport-related genes. We identified a set of the genes from the gill transcriptome from a freshwater crayfish *Cherax quadricarinatus* using the Illumina NGS-sequencing technology. We identified and characterized carbonic anhydrase (CA) genes and some other key genes involved in systematic acid-base balance and osmotic/ionic regulation. We also examined expression patterns of some of these genes across different sublethal pH levels [1]. A total of 72,382,710 paired-end Illumina reads were assembled into 36,128 contigs with an average length of 800 bp. About 37% of the contigs received significant BLAST hits and 22% were assigned gene ontology terms. These data will assist in further physiological-genomic studies in crayfish.

## Specifications table

**Table:** 

Subject area	Biology
More specific subject area	RNA-seq transcriptome data of crayfish (*Cherax quadricarinatus*)
Type of data	Table and figures
How data was acquired	Sequencing with Illumina HiSeq 2000
Data format	Raw and analyzed
Experimental factors	Samples were exposed to three pH levels (6, 7 and 8)
Experimental features	Gills from three pH levels were pooled. RNA was extracted from the pooled samples using TRIZOL/Chloroform method and purified further with a RNeasy Midi Kit (cat # 75144, QIAGEN). RNA yield and quality were checked using a 2100 RNA nanochip at bioanalyzer. RNA was sequenced with Illumina HiSeq 2000.
Data source location	Cherax park Aquaculture, RMB 694 Kanyan Rd, Theebine QLD 4570, Australia
Data accessibility	Analyzed data is with this article and raw sequence data was deposited in the NCBI Sequence Read Archive under the accession number.

## Value of the data

• It provides valuable information on the gill expressed carbonic anhydrase genes and some other key genes involved in pH and salinity balance.
• Scientists and researchers will be able to access and utilize the RNA-seq data through the link.
• It facilitates the scientists to further genomics and physiological studies in crayfish.
• The data can be used as reference gill transcriptomes for the freshwater crayfish (*Cherax quadricarinatus*).

## 1 Data, experimental design, materials and methods

### 1.1 Data description

**Fig. 1:**
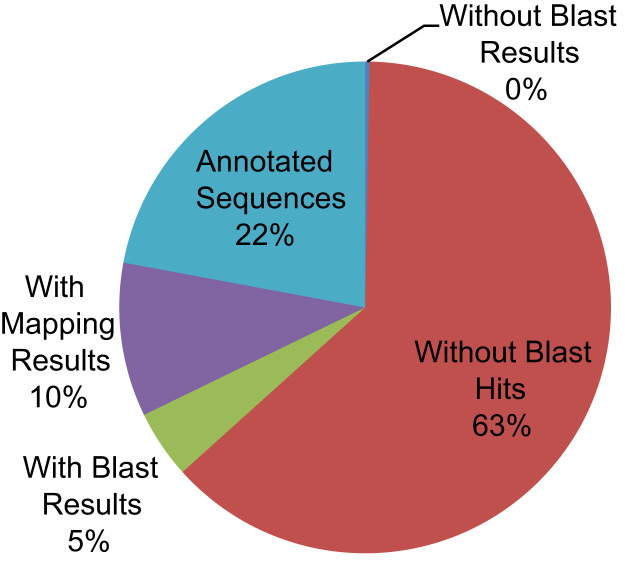
Data distribution of the assembled contigs after blasting, mapping and annotation.

**Fig. 2:**
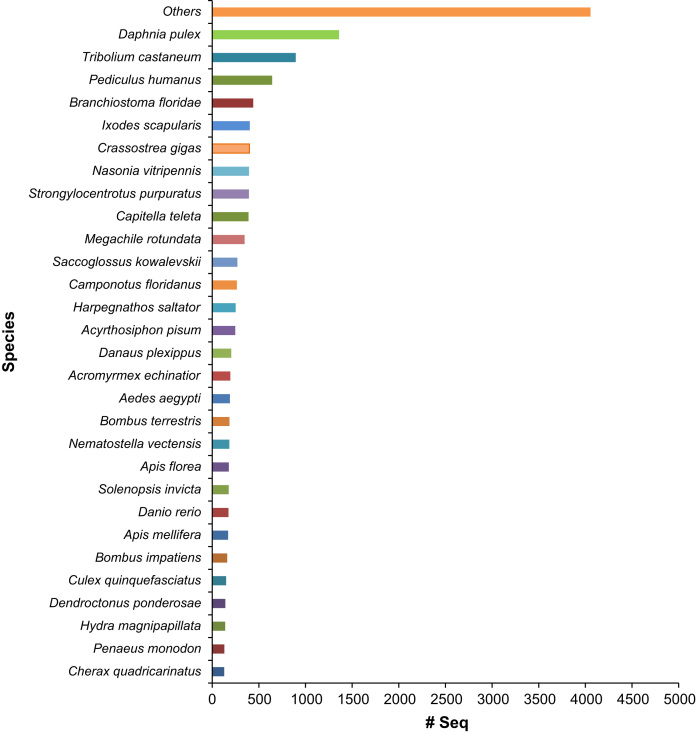
Major top-hit species distribution on the basis of BALST search using the assembled contigs.

**Fig. 3:**
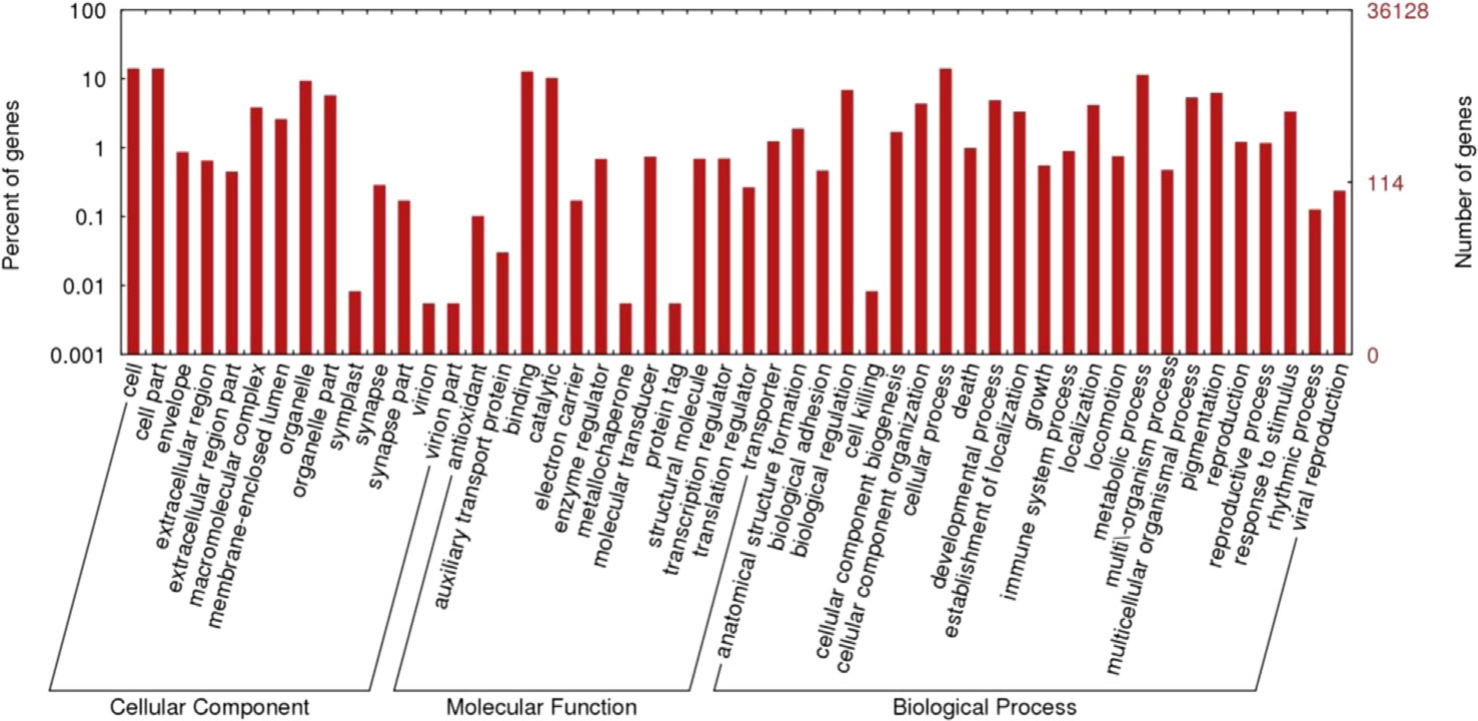
Gene Ontology distribution of the gill transcripts from *Cherax quadricarinatus* based on biological processes, molecular functions and cellular components.

**Fig. 4:**
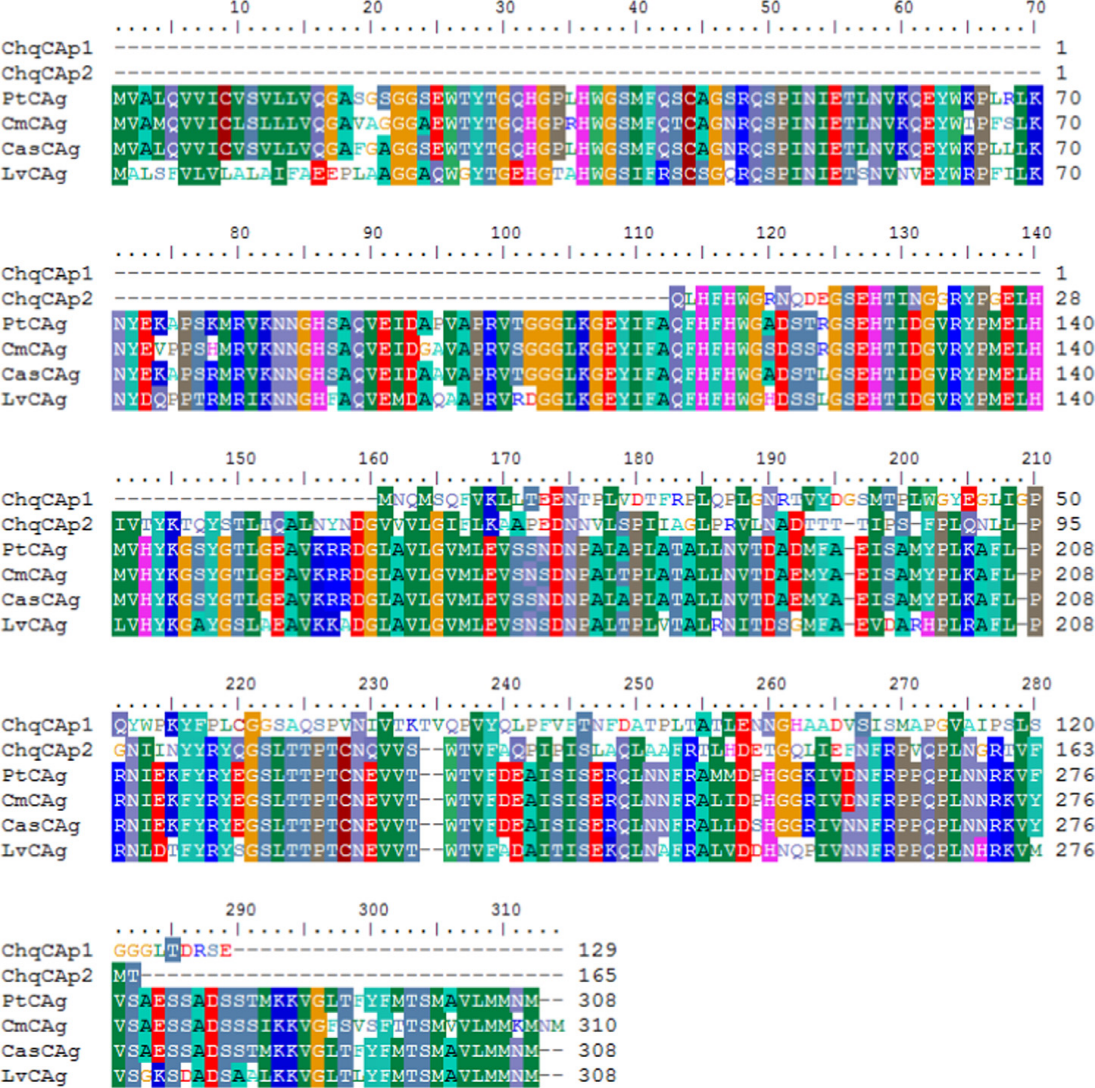
Multiple alignment of translated amino acid sequences of two partial *C. quadricarinatus* CAs (ChqCAp1: KM610228 and ChqCAp2: KM880150) with CA isoforms from some representative crustaceans such as the pacific white shrimp *Litopenaeus vannamei* (LvCAg: AGC70493), the littoral crab *Carcinus maenas* (CmCAg: ABX71209), the horse crab *Portunus trituberculatus* (PtCAg: AFV46145) and the blue crab *Callinectes sapidus* (CasCAg: ABN51214). The letter g after CA indicates the Glycosyl-phosphatidylinositol-linked carbonic anhydrase.

Through the sequencing of *Cherax quadricarinatus* gill transcriptome library, we obtained over 72 million (72,382,710) good quality paired-end sequence reads (90 bb each) after the removal of low quality reads. The sequenced data was deposited in NCBI Sequence Read Archive under the accession number PRJNA275170. Illumina sequence reads were assembled in 36,128 contigs. Average contig length was 800 bp, the N50 was 936 bp and the longest contig was 14,972 bp (see Table 1). Approximately 37% of the contigs received significant BLAST hits and 22% were assigned gene ontology terms (see Fig. 1). Arthropod species represented the majority of top BLAST hits (76% of the top 30 Species) (see Fig. 2). Approximately 48.5%, 29.2% and 22.3% of the GO terms were assigned to Biological Processes, Molecular Function and Cellular Component GO categories, respectively (see Fig. 3). Three full length CA isoforms; cytoplasmic CA, glycosyl-phosphatidylinositol-linked CA, and β-CA as well as two partial CA gene sequences were identified. All the CA isoforms showed high protein-similarity with other decapod crustaceans (see the protein alignment results of the partial CDs of CAg in Fig. 4 and those of the full CDs in Ref [1]). Expression patterns of the CAs, Na^+^/K^+^-ATPase, V-type H^+^-ATPase and Arginine kinase were examined at pH 6, 7 and 8. Only the cytoplasmic CA gene (KM538165) showed significant differences in expression across different pH levels [see in Ref: [1]].

## 2 Materials and methods

### 2.1 Animal collection and preparation

Live crayfish (*C. quadricarinatus*) were collected from a crayfish farm situated at Theebine QLD, Australia. Animals were reared in rectangular glass tanks (27 L-capacity each) and acclimated for 2 weeks at temperature 27 °C, pH 8 and conductivity 500 μS/cm before the experiment started. During the acclimation period all animals were fed regularly with formulated feed pellets. Water quality was maintained at temperature 27–28 °C, pH 7.5 and conductivity 450–550 μS/cm with a computer-controlled filtration system. Feeding was stopped 24 h before the treatment and the animals were distributed into three separate glass tanks (25×18×15 cm^3^) with water temperature set at 25.5±0.9 °C and conductivity 521.8±29 (μS/cm). A total of nine individuals (length 131.9±6 mm and body weight 56.3±6 g) were used for the experiment. These animals were placed in three different pH levels (6, 7 and 8), a treatment within the tolerance range of this species [3,4]. Gill tissue was extracted at 3th, 6th and 12th hours.

### 2.2 RNA extraction and sequencing

Animals were euthanized in crushed ice for 5–10 min before the tissue extraction. Gill tissue were dissected and immediately stored in RNA*later* solution (Life technologies). Tissues preserved in RNA*later* were stored at −80 °C prior to RNA extraction. Total RNA was extracted from the pooled gill tissues, which contained equal amounts of sample from each of the animals from all treatments using a TRIZOL/Chloroform extraction [2] and then purified further using a RNeasy Midi Kit (cat # 75144, QIAGEN). RNA yield and quality were checked using agarose gel electrophoresis and a bioanalyzer using a 2100 RNA nanochip. RNA was sequenced at the Beijing genomics institute and prepared using the same protocol as described in [5].

### 2.3 Data assembly and annotation

Illumina paired end sequences were assembled into contigs using CLC Genomics Workbench (version 6.0.2). The assembled data were blasted, mapped and annotated using Blast2Go Pro software [6]. Sequence annotation information was retrieved for sequences that had BLASTx queries exceeding a stringency of e-value<10^−5^. The statistics on data distribution and top-hit species distribution were obtained using the analysis tool in Blast2Go Pro (see Figs. 1 and 2). For contigs that received significant BLAST hits with protein function information, Gene Ontology (GO) terms were assigned and their distribution among GO categories was mapped using WEGO [7] (see Fig. 3). Enzyme Commission numbers were assigned and the relevant maps from the KEGG (Kyoto Encyclopedia of Genes and Genomes) were downloaded in order to predict the metabolic pathways for each contig [8].

### 2.4 Identification of candidate genes

The transcriptome data set was screened to identify transcripts matching CA genes and other targeted osmoregulatory genes. The identified gene sequences were aligned with the non-redundant protein database at NCBI in order to compare the similarity to previously identified and annotated genes. Following this validation step, 3′ and 5′-untranslated regions (UTR) as well as open reading frames were determined using ORF Finder at NCBI [see accession in Table 2 in Ref: [1]]. The potential cleavage site of the signal peptide was predicted using PrediSi [9] and N-linked glycosylation sites were predicted with N-GlycoSite [10] using NXS/T model (where N=Aspargine, S=Serine, T=Theronine and X=any amino acid). Open reading frames were translated into proteins. The putative protein domains of the open reading frames was analyzed using SMART (Simple Modular Architecture Research Tool) database [11]. The translated amino acids sequences were used as BLASTp queries against the NCBI database. Up to 20 top BLAST hits were downloaded for each gene and aligned with the *C. quadricarinatus* candidate gene in BioEdit Sequence Alignment Editor (version 7.2.5; [12]) using a ClustalW alignment platform [13]. For the full-length CA genes Neighbor Joining trees were generated in Geneious (version 8.0.4; [14]) using the Jukes Cantor method with 1000 bootstraps.

### 2.5 Data validation and expression study with RT-PCR

Live crayfish were exposed to three pH treatments, pH 6, 7 and 8, which are in a similar range in natural habitats [3,4]. Animals were harvested after 24 h and gills were extracted immediately after animals were euthanized in crushed ice for 5–10 min. Three replicates of animals were used in each experiment. Specific quantitative real-time PCR primers were designed for transcripts that identified as the three CA genes, V-type H^+^-ATPase, Arginine kinase and 18s rRNA based on the transcripts obtained in our study [see Table 1 in Ref. [1]]. Realtime PCR conditions were maintained as: pre-incubation of 95 °C for 5 min, followed by a total 45 cycles of three-step amplification of 95 °C for 10 s; 60 °C for 10 s and 72 °C for 10 s using a LightCycler 96 RT-PCR machine and reagents (Roche, Version 04, Cat. no. 06924204001). Ribosomal 18S was used as an internal control gene to normalize sample-to-sample variations. The relative expression of the target genes were measured as a ratio (Ratio=concentration of target gene/concentration of 18S gene) using Relative Quant analysis tool described in the Light Cycler 96 system operator׳s guide, version 2.0.

#### 2.5.1 Direct link to deposited data

Raw sequence data was deposited in the SRA (Short Read Archive) repository of NCBI under the accession number PRJNA275170. Data can be downloaded through the link: http://www.ncbi.nlm.nih.gov/sra/?term=PRJNA275170

## Figures and Tables

**Table 1:** Summary statistics for assembled contigs generated from *C. quadricarinatus* gill transcriptomes.

**Statistics**	**bp/number**
Total number of contigs:	36,128
N50	936 bp
N75	535 bp
N25	1835 bp
Mean contig length	800 bp
Minimum contig length	283 bp
Length of the longest contig	14,972 bp
Number of contigs longer than 500 bp	20,554
Number of contigs longer than 1500 bp	3807
GC content	43%
